# The American Medical Association

**Published:** 1856-04

**Authors:** 


*The American Medical Association.*

The next annual meeting of this Association will commence in Detroit, on Tuesday, the 6th day of May. The Secretary has given notice that the meetings will be held in Fireman's Hall, where Delegates can call to obtain information on their arrival in the city. From present indications, we think the profession of Illinois, and the North-West generally, will be well represented. Let no delegate remain at home from trifling causes.

